# The “Decline and Fall” of Nontyphoidal *Salmonella* in the United Kingdom

**DOI:** 10.1093/cid/cis967

**Published:** 2012-11-19

**Authors:** Sarah J. O'Brien

**Affiliations:** University of Liverpool Institute of Infection and Global Health, National Consortium for Zoonosis Research, Neston, United Kingdom

**Keywords:** *Salmonella*, eggs, vaccination, food safety, public health

## Abstract

Remarkable changes in the epidemiology of human nontyphoidal salmonellosis have occurred in the United Kingdom over the last century. Between 1981 and 1991, the incidence of nontyphoidal salmonellosis in the United Kingdom rose by >170%, driven primarily by an epidemic of *Salmonella enterica* subspecies *enterica* serovar Enteritidis phage type (PT) 4, which peaked in 1993. Measures introduced to control this epidemic included legislation, food safety advice, and an industry-led vaccination program in broiler-breeder and laying poultry flocks. The incidence of *Salmonella* Enteritidis has been falling since 1997, and levels of *Salmonella* Enteritidis PT4 have fallen to preepidemic levels and have stayed low. The temporal relationship between vaccination programs and the reduction in human disease is compelling and suggests that these programs have made a major contribution to improving public health.

## Abstract (precis)

A remarkable decline in nontyphoidal salmonellosis in the United Kingdom since the late 1990s coincides closely with the introduction of voluntary vaccination programs in broiler-breeder and laying flocks, demonstrating the success of this concerted, industry-led public health action.

Nontyphoidal *Salmonella* species are important foodborne pathogens worldwide [1], causing diarrhea, vomiting, nausea, fever, and abdominal pain. Illness has been linked to a wide range of food items including eggs, chicken, beef, pork, salad vegetables, and dairy products, and other risk factors including overseas travel [2–7]. Outbreaks are fairly common [5]. The burden of illness, defined as morbidity and mortality, is substantial. In the United States, nontyphoidal *Salmonella* species are estimated to cause 1 million foodborne illnesses [8] and are the leading cause of death among foodborne bacterial pathogens [9]. Across the 27 member states of the European Union (EU), there were estimated to be 6.2 million cases of salmonellosis in 2009 [10]. In a population-based study in the United Kingdom (UK) in 2008–2009, there were >38 600 estimated cases and nearly 11 300 patients presenting to a primary care physician [11]. This represented a marked reduction in incidence compared with a similar study conducted more than a decade earlier [12, 13]. The purpose of this article is to discuss the factors associated with a substantial decline in nontyphoidal salmonellosis in the United Kingdom since the mid-1990s.

## A BRIEF HISTORY OF NONTYPHOIDAL SALMONELLOSIS IN THE UNITED KINGDOM

Remarkable changes in the epidemiology of human nontyphoidal salmonellosis have occurred in the United Kingdom over the last century. Prior to 1942, the dominant foodborne salmonellas causing disease were *Salmonella enterica* subspecies *enterica* serovar Typhimurium, *Salmonella* Enteritidis, *Salmonella* Thompson, *Salmonella* Newport, *Salmonella* Bovismorbificans, and *Salmonella* Choleraesuis [14]. *Salmonella* Typhimurium remained the dominant serovar causing human disease for much of the 20th century, although there were fluctuations in other salmonellas in the “top 10” over time. For example, *Salmonella* Agona emerged as an important serovar in the 1960s following its introduction into pigs and poultry through contaminated fish meal imported from Peru [15]. *Salmonella* Hadar became the second most commonly isolated cause of human nontyphoidal salmonellosis in the mid-1970s when particular genetic lines of turkeys became infected [15]. Against this background, the incidence of *Salmonella* Enteritidis increased fairly gradually from around 150 to approximately 900 laboratory-confirmed cases per year between 1961 and 1980 [16]. During this time, phage type (PT) 8 dominated and was responsible for several turkey-associated outbreaks in the late 1960s [16]. By 1975 *Salmonella* Enteritidis was consistently the second or third most frequently isolated serovar annually [17].

**Figure 1.**
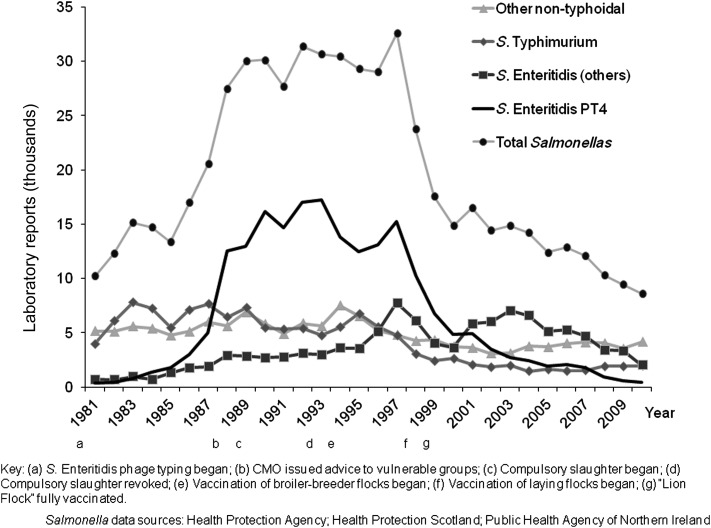
Laboratory reports of human *Salmonella* cases in the United Kingdom, 1981–2010. Abbreviations: CMO, Chief Medical Officer; PT, phage type.

Between 1981 and 1991, the incidence of nontyphoidal salmonellosis in the United Kingdom rose by >170% [18], driven primarily by an epidemic of *Salmonella* Enteritidis PT4 [16, 18–20] (Figure 1). In 1981 *Salmonella* Enteritidis accounted for approximately 10% of human *Salmonella* illnesses, but by 1993 this proportion had risen to nearly 70% [20]. In the early 1980s, PT4 overtook PT8 to become the predominant phage type in 1983, comprising 46% of isolations that year. By 1988 PT4 had risen to account for 81% of *Salmonella* Enteritidis strains isolated [16] and had ended the political career of a prominent government minister [21]. The United Kingdom was not alone; analysis of data submitted to the World Health Organization's *Salmonella* surveillance system showed that *Salmonella* Enteritidis in the late 1980s was increasing on several continents, with North America, South America, and Europe appearing to bear the brunt [22].

## EVIDENCE THAT THE DECLINE IN *SALMONELLA* IS REAL

Compelling evidence that the decline in *Salmonella* is real is derived from 3 sources. The first comprises 2 population-based prospective cohort studies of infectious intestinal disease (IID) in the community conducted more than a decade apart [11–13]. The primary outcome measures in both studies were estimates of the incidence of IID in the community, presenting to primary healthcare and reported to national surveillance. They were conducted using identical study designs and case definitions and employed similar microbiological methods, the exception being that molecular microbiological techniques were used alongside traditional microbiology in the second study of infectious intestinal disease (IID2). In the first study of infectious intestinal disease (IID1) in 1993–1996, the incidence of nontyphoidal *Salmonella* in the community in England was 2.2 cases per 1000 person-years (95% confidence interval [CI], 1.1–4.3) but by 2008–2009 this had fallen to 0.7 cases per 1000 person-years (95% CI, .2–3.0). For nontyphoidal *Salmonella* cases presenting to primary care in England, the incidence rate had fallen from 1.6 cases per 1000 person-years (95% CI, 1.2–2.1) in IID1 to 0.2 cases per 1000 person-years (95% CI, .1–.5) in IID2. The decline in incidence in the community was not statistically significant because in IID2 the study power was insufficient to detect statistically significant changes in organism-specific incidence—to do this would have required >100 000 person-years of follow-up, based on incidence rates in IID1. Nevertheless, the reduction in presentations to primary healthcare was statistically significant.

Second, there has been a substantial fall in laboratory-confirmed *Salmonella* cases reported to national surveillance (Figure 1). Phage typing of *Salmonella* Enteritidis was implemented from 1981 as an addition to the centralized, national service already in existence for confirmation and further typing [17], and all clinical diagnostic laboratories have continued to refer all *Salmonella* isolates to the national reference laboratories since that date. At the beginning of 1992, 2 separate national *Salmonella* databases were merged to form a single national dataset, which became patient-based rather than isolate-based, thus eliminating potential duplication if people were tested more than once [18]. Laboratory testing methods have remained constant since then and reporting algorithms have not changed [23], suggesting that the reduction in *Salmonella* is real. When *Salmonella* Enteritidis PT4 peaked in 1993 in the United Kingdom, >18 000 laboratory-confirmed cases of illness were recorded in national surveillance statistics, yet by 2010 PT4 isolations had fallen to just 459 [24]. Thus, the decline in nontyphoidal salmonellosis witnessed in the United Kingdom in recent years reflects this major contraction in reports of *Salmonella* Enteritidis PT4.

Finally, outbreaks of salmonellosis have declined. Standardized reporting of outbreaks of gastrointestinal infection was introduced in 1992 in England and Wales and in 1996 in Scotland partly in response to the increase in nontyphoidal salmonellosis. A foodborne outbreak is defined in European legislation as “an incidence, observed under given circumstances, of two of more human cases of the same disease and/or infection, or a situation in which the observed number of human cases exceeds the expected number and where the cases are linked, or are probably linked, to the same source” [25]. Between 1992 and 2008, foodborne *Salmonella* outbreaks reported to national surveillance fell from nearly 150 per year to just over 20 annually, and the pattern of decline closely mirrors that of laboratory-confirmed cases [25].

## EPIDEMIOLOGY OF *SALMONELLA* ENTERITIDIS IN THE UNITED KINGDOM

Epidemiologic investigations of outbreaks and sporadic cases repeatedly showed that *Salmonella* Enteritidis PT4 infection in humans was frequently associated with consumption of poultry meat and hens' eggs on both sides of the Atlantic [25–31]. In nearly 2500 foodborne disease outbreaks reported to the UK Health Protection Agency between 1992 and 2008, *Salmonella* species accounted for 47% of all outbreaks, 46% of cases, 70% of hospital admissions, and 76% of deaths [25]. *Salmonella* Enteritidis PT4 was the causative organism in 51% of all the *Salmonella* outbreaks throughout the surveillance period but the percentage of outbreaks caused by *Salmonella* Enteritidis PT4 declined from the late 1990s onward. At least one food vehicle was identified in 75% of outbreaks reported, and poultry meat was the vehicle most often implicated (19% of outbreaks). Desserts were also implicated commonly (11% of outbreaks), and raw shell eggs were used as an ingredient in 70% of these desserts. Eggs were implicated separately in an additional 6% of outbreaks. Analysis of outbreak data also showed that nearly 50% of foodborne *Salmonella* outbreaks occurred in the food service/catering sector.

*Salmonella* Gallinarum and *Salmonella* Pullorum had been the dominant *Salmonella* serovars in UK poultry until the early 1970s. These strains both caused clinical disease in the birds and were virtually eradicated by a combination of slaughtering of seropositive hens and vaccination [20]. However, the ecological niche left by these 2 serovars was filled by *Salmonella* Enteritidis. Complete genome sequencing of a host-promiscuous *Salmonella* Enteritidis PT4 isolate (P125109) and a chicken-restricted *Salmonella* Gallinarum isolate (287/91) has indicated that *Salmonella* Gallinarum 287/91 is a recently evolved descendent of *Salmonella* Enteritidis [32]. Importantly, *Salmonella* Enteritidis infects poultry without causing overt disease, which probably facilitated its rapid spread internationally [20]. Another key feature of *Salmonella* Enteritidis is colonization of the reproductive tissues leading to the production of eggs with *Salmonella*-positive contents [20, 33] and, in some eggs, the numbers of organisms can be very high [34].

## CONTROLLING SALMONELLOSIS AND OTHER FOODBORNE ILLNESSES

In August 1988, as evidence of a link between *Salmonella* Enteritidis PT4 and raw shell eggs strengthened, the Chief Medical Officer issued advice to consumers to avoid eating raw eggs or uncooked foods in which raw eggs were an ingredient. In December of the same year, he issued further advice to vulnerable people such as the elderly, individuals with chronic illness, infants, and pregnant women. They were counseled only to eat eggs that had been cooked until the yolks and whites were solid [18]. Caterers were encouraged to use pasteurized eggs, especially where foodstuffs were not going to be cooked further (eg, mayonnaise), and it was recommended that eggs be considered short shelf-life products. They should be refrigerated <8°C throughout the production chain and during retail, catering, and domestic storage, and consumed within 3 weeks of the date of lay [18]. In 1989 the government introduced a raft of legislation, including the Zoonoses Order, which required that all *Salmonella* isolates from live animals or birds, carcasses, or feedstuffs be reported. Movement restrictions were implemented along with compulsory slaughter, compensation, and disinfection procedures. The more draconian procedures were usually reserved for *Salmonella* Typhimurium and *Salmonella* Enteritidis [24]. The requirement for compulsory slaughter of poultry flocks was revoked following a recommendation from the Advisory Committee on the Microbiological Safety of Food in 1993 to review the policy in light of the fact that *Salmonella* Enteritidis in flocks had reduced substantially [18]. In 1989, >600 000 birds from 58 infected flocks were slaughtered. In 1992, <300 000 birds from 38 infected flocks were slaughtered [18]. Alongside legislation was a voluntary, industry-led vaccination scheme that began in broiler-breeder flocks in 1994 and in laying flocks in 1998 [16]. A “Lion Mark,” stamped on eggs, which had been introduced in 1957 but dropped by 1971, was revived in 1998 (http://www.lioneggs.co.uk/page/lionmark). The Lion Mark can only be used by subscribers to the British Egg Industry Council for eggs that have been produced in accordance with UK and EU law and the Lion Quality Code of Practice. The code of practice requires mandatory vaccination of all pullets destined to lay Lion eggs against *Salmonella*; independent auditing; full traceability of hens, eggs, and feed; and a “best-before” date stamped on the shell and pack, in addition to on-farm stamping of eggs and packing station hygiene controls.

When, in 1989, a Junior Health Minister stated in a British television interview that “Most of the egg production in this country, sadly, is now infected with *Salmonella*,” the sale of eggs collapsed by 60% almost overnight. Moreover, despite government efforts to improve the safety of eggs, sales continued to fall by around 8% per year over the next 10 years, which was a disaster for the industry. The British Egg Industry Service began a major consumer research program in 1997 and, in 1998, the majority of UK producers and packers made a voluntary investment of £8 million to assist the British Egg Industry Council to relaunch British eggs. A total of £4 million was spent on the stringent new Code of Practice described above, and £4 million supported a new promotional campaign to restore consumer confidence and increase consumption. The cost of the vaccination program (including Lion sampling and testing) is estimated to be around £52 million to date (Mark Williams, written personal communication, September 2012). However, between 1998 and 2009, the egg market grew from 9.8 billion to 11 billion eggs per year, and Lion eggs now account for around 85% of the total market. Within the retail sector the market share of Lion eggs share rose from approximately 60% in 1998 to 95% in 2010 (http://www.lioneggs.co.uk/files/lioneggs.co.uk/pdfs/marketing.pdf).

Alas, *Salmonella* was not the only “food scare” during the 1980s and 1990s. Scandals surrounding, for example, bovine spongiform encephalopathy in the United Kingdom, dioxins in Belgium, and *Salmonella* EU-wide prompted new legislation providing for a risk-based “farm to fork” approach to food safety policy, which was enacted in 2002 (European General Food Law [Regulation (EC) No. 178/2002]) [24]. EU Zoonoses Regulation (EC) No. 2160/2003 required member states to take effective measures to detect and control *Salmonella* species of public health significance in specified animal species at all relevant stages of production [24]. Each EU member state was obliged to undertake a standardized baseline survey to determine the prevalence of *Salmonella* within their industry sectors. EC Regulation (EC) 1168/2006 laid down an annual reduction target for *Salmonella* Enteritidis and *Salmonella* Typhimurium for each member state.

## NATIONAL CONTROL PROGRAMS FOR *SALMONELLA* IN THE POULTRY SECTOR

Four National Control Programmes (NCPs) for *Salmonella* have been implemented in the UK poultry sector between 2007 and 2010. These postdate the rapid decline in *Salmonella* Enteritidis in the United Kingdom but are designed to achieve and maintain low rates EU-wide. For the most part, the targets set by the EU have already been met or exceeded in the United Kingdom [24].

1. The NCP for breeding chickens (implemented in 2007): The target for this NCP was that no more than 1% of adult breeding flocks should be infected with 5 specific regulated serovars (*Salmonella* Enteritidis, *Salmonella* Typhimurium, *Salmonella* Hadar, *Salmonella* Infantis, and *Salmonella* Virchow) by the end of 2009. Results from UK holdings have been significantly below the EU target of 1% every year for the last 4 years [24].
2. The NCP for commercial laying chickens (implemented in 2008): An EU-wide baseline survey of commercial laying chicken flock holdings was undertaken in 2004–2005. In a survey of *Salmonella* species on 454 commercial layer flock holdings in the United Kingdom, 54 (11.7%) were *Salmonella* positive [35]. *Salmonella* Enteritidis was the serovar most commonly identified (prevalence = 5.8%) and PTs 4, 6, 7, and 35 comprised 70% of isolates. *Salmonella* Typhimurium was the second most commonly identified serovar (prevalence = 1.8%). The UK prevalence figures were among the lowest of the major egg-producing countries (7.9% of holdings positive compared with a 20.4% average across the EU) [36]. Across the EU, the incidence rate of salmonellosis in member states varies between 16 and 11 800 per 100 000 population and has been shown to be significantly correlated with the prevalence of *Salmonella* Enteritidis in laying hens [10], so controlling levels of *Salmonella* Enteritidis in laying flocks is important for improving public health.
3. The NCP for broilers (implemented in 2009): The target for this NCP was that no more than 1% of flocks should be infected with *Salmonella* Enteritidis and *Salmonella* Typhimurium by the end of 2011. In a baseline survey of broiler chickens in 2005–2006 in the United Kingdom, the prevalence of *Salmonella* Enteritidis and *Salmonella* Typhimurium was very low (0.2% [37] compared with an EU average of 11.0% [38]) and remains well below the EU target [24].
4. The NCP for turkeys (implemented in 2010): A baseline survey for *Salmonella* in turkey breeding and fattening flocks was carried out across the EU in 2006–2007. In the United Kingdom, the prevalence of *Salmonella* in breeding flock holdings was 20.1% and in fattening flocks the holdings prevalence was 37.7% [39]. The flock prevalence of *Salmonella* Typhimurium was very low on breeding holdings at 0.7% (EU weighted average = 1.8%) but higher on fattening holdings at 4.6% (EU weighted average = 3.7%) [24]. The target for *Salmonella* reduction is that only 1% of breeding flocks and 1% of fattening flocks should be positive by the end of 2012. Early indications are that this target will be met.

## WHAT NEXT?

There is no room for complacency. During the 2000s, new *Salmonella* problems emerged. Notable among these were national outbreaks of *Salmonella* Enteritidis PT14b linked to raw shell eggs originating in Spain [40, 41]. Unbelievably, perhaps, hospital caterers in the United Kingdom were found serving raw shell eggs again to patients, with consequent outbreaks [42]. The first outbreak of *Salmonella* Typhimurium PT8 linked to consumption of duck eggs since 1949 occurred in the United Kingdom [43], and *Salmonella* outbreaks linked to fresh produce were increasingly recognized [44, 45], reflecting a pattern also seen in the United States [46].

## CONCLUSIONS

The nature of public health interventions often means that evaluating their impact is complex as they are often implemented in combination and/or simultaneously. It is interesting to reflect on the fact that the various legislative measures in the United Kingdom in the late 1980s and early 1990s appear to have slowed down the increase in *Salmonella* Enteritidis PT4, whereas the decrease in laboratory-confirmed human cases coincides quite closely with the introduction of vaccination programs in broiler-breeder and laying flocks and prior to much of the EU legislation being implemented. It is probable that no single measure contributed to the decline in *Salmonella* Enteritidis PT4 and that the combination of measures was successful, but the temporal relationship between vaccination programs and the reduction in human disease is compelling and suggests that these programs have made a major contribution to improving public health.

There has also been a reduction in reported human salmonellosis cases across the EU (on average 12% per year between 2005 and 2009). The European Commission and European Food Safety Authority are attributing this, at least in part, to successful control of *Salmonella* in broiler, laying, and breeding hen flocks and eggs [24].

If success in public health is defined by illnesses averted, then the story of *Salmonella* Enteritidis PT4 in the United Kingdom, which has come down and stayed down, is good news. However, history teaches us that something else may come along to take its place. Robust surveillance, incorporating state-of-the-art microbiological, epidemiological, and biostatistical methods, and maintaining a prompt and comprehensive response to outbreaks is just as important now as it ever was.
